# Evaluating the risk of transfusion and transplant‐transmitted monkeypox infections

**DOI:** 10.1111/tme.12918

**Published:** 2022-09-22

**Authors:** Heli Harvala, Peter Simmonds

**Affiliations:** ^1^ Microbiology Services NHS Blood and Transplant London UK; ^2^ Nuffield Department of Medicine University of Oxford Oxford UK

**Keywords:** blood transfusion, monkeypox, monkeypox virus, nucleic acid testing, poxviridae, transmission, transplant

## Abstract

The recent emergence of monkeypox virus (MPXV) in the UK and elsewhere is of urgent public health concern. Several aspects of MPXV epidemiology and pathogenesis, including its systemic spread and viraemia during acute infection, furthermore represent an important potential threat to the safety of blood transfusion and organ transplantation. Reported infections in the UK have been exponentially increasing over the last 2 months, with 1552 reported cases in the UK by 7th July 2022. This is likely to be considerable underestimate given current limitations in diagnostic capacity and clinical diagnoses hampered by its similar disease presentations to other causes of rash and genitourinary disease. While MPXV infections are currently most widespread in gay, bisexual or other men who have sex with men, wider spread of MPXV outside defined risk groups for infection may prevent identification of infection risk in donors. While typically mild disease outcomes have been reported in UK cases, case fatality rates ranging from 1% to over 10% are reported for different MPXV strains in its source area in sub‐Saharan Africa. Recipients of blood components and organs transplant, especially those who are immunosuppressed, may reproduce the greater systemic spread and morbidity of those infected through percutaneous routes. There is a potential risk of MPXV transmission and severe disease outcomes in blood and transplant recipients. In addition to current risk assessments performed in the UK and exclusion of donors with recent MPXV exposure, determining viraemia frequencies in donors and directly evaluating transmission risk would be of considerable value in assessing whether MPXV nucleic acid screening should be implemented.

## 1 INTRODUCTION

**FIGURE 1:**
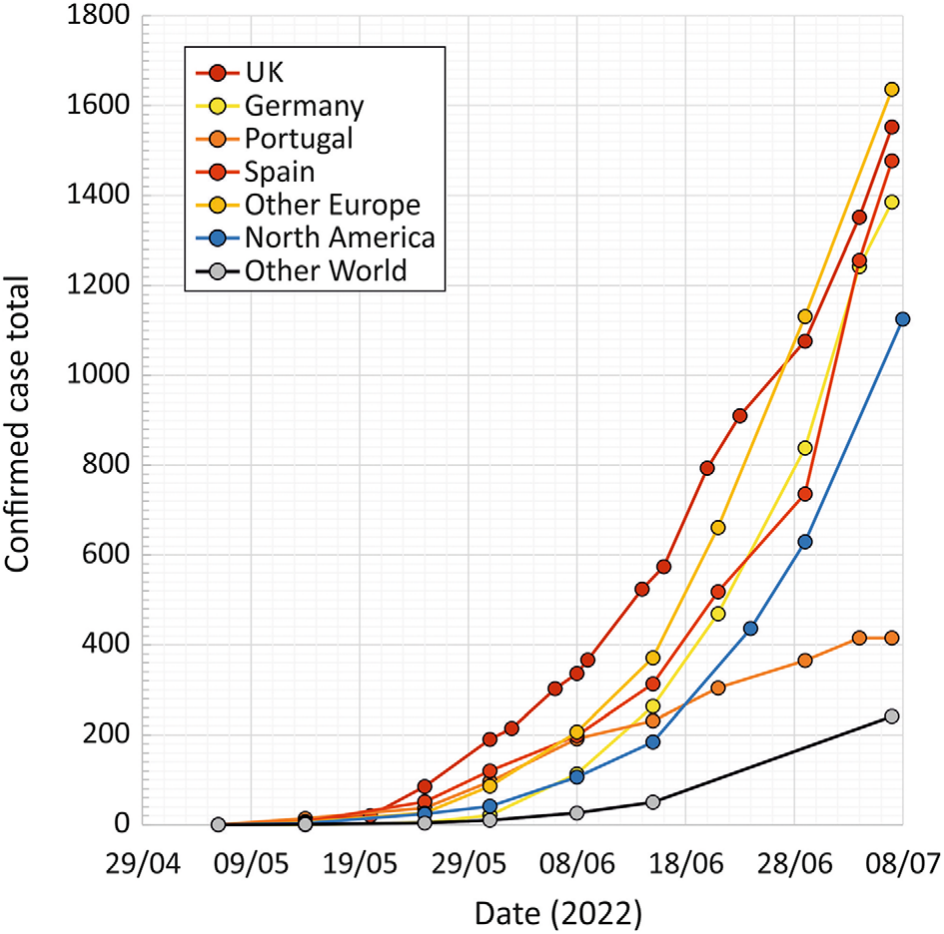
Totals of confirmed cases of MPXV in UK, selected European countries and totals of other regions since its emergence in early May. Data sources: ECDC, UKHSA, CDC, (https://www.ecdc.europa.eu/en/monkeypox‐outbreak; https://www.gov.uk/government/publications/monkeypox‐outbreak‐epidemiological‐overview; https://www.cdc.gov/poxvirus/monkeypox/response/2022/world‐map.html)

There have been a multitude of reports of monkeypox (MPX) in the United Kingdom, several countries in Europe, North America and increasingly worldwide over the last 2 months (Figure 1). Reported cases, initially described in the UK at the beginning of May have dramatically increased in number with a cumulative total of 1552 at the time of writing (10th July), and an estimated doubling time of 15 days (95% confidence interval: 10–18 days).^1^ Exponentially increasing number of cases have also been reported in several European countries, in North America and elsewhere (Figure 1). MPX virus (MPXV) infections have been preferentially detected in males (all but 4 of currently reported UK cases), primarily in those aged between 20 and 49, and preferentially infecting gay, bisexual, or other men who have sex with men (GBMSM).

Interim case definitions for MPX has been proposed by the UK Health Security Agency (UKHSA) and European Centre for Disease Prevention and Control (ECDC),^2^, ^3^ but MPXV infections remain problematic to diagnose clinically, often presenting with a single lesion only or with lesions that may be mistaken for varicella zoster or where affecting genital areas, those caused by sexually transmitted diseases such as syphilis. Access to diagnostic testing for MPXV DNA is highly limited at the time of writing and may delay definitive diagnoses from being made in many cases. Although relevant professional associations and sexual health clinics across Europe have been contacted with information about MPXV infections and presenting symptoms, an informed clinical diagnosis is difficult as MPXV may present clinically similarly to other sexually transmitted infections. Finally, infections may be mild, present with atypical symptoms or be entirely asymptomatic and hence remain undiagnosed. Collectively, it is almost certain that the extent of MPXV spread is currently substantially under‐estimated across Europe.

MPXV infections are associated with systemic spread and a prolonged period of viraemia in pre‐symptomatic and symptomatic stages of infection.^4^ MPXV may therefore potentially be transmitted by blood components and organ transplantation. In this review, we evaluate current transmission risk in a transfusion or transplant setting, determine what steps are required to evaluate the extent of its emergence and how transmissions may be prevented in the short term.

## 2 BACKGROUND

MPXV is one of 12 species of poxviruses classified in the *Orthopoxvirus* genus of the virus family, *Poxviridae*.^5^ Poxviruses form large rectangular virions (250–450 nm) that show a complex non‐isometric symmetry. They possess large DNA genomes that are replicated in membranous structures in the cytoplasm and encode several structural, replicative and moreover an extremely large and highly variable complement of “accessory” genes that enable the virus to evade cellular and adaptive immune responses to infection.^6^ Poxviruses are typically exquisitely adapted to their hosts, partly through the specificity of their evasion mechanisms but with variable relationships between transmissibility and pathogenicity, smallpox of humans representing one end of a continuum that extends to clinically inapparent infections with vaccinia virus (buffalopox) at the other.

MPXV was first recognised as a separate entity from smallpox and other poxviruses in the late 1950ss/early 1960s^7^, ^8^ and currently represents the most widely distributed orthopoxvirus to infect humans following the eradication of smallpox by vaccination.^9^, ^10^, ^11^ MPXV has been detected in the wild in the rope squirrel, the tree squirrel and the Gambian pouched rat in central Africa^12^, ^13^, ^14^ with serological evidence for a wider distribution in sub‐Saharan rodents.^15^ Rodents may represent a source of zoonotic infections of humans and other primates in Central and West Africa.^16^, ^17^ The route of zoonotic transmission to humans is not well characterised, although MPXV is not generally considered to be easily transmitted between humans.^18^ The outbreak of MPXV infection in the USA in 2003^19^, ^20^, ^21^ is illustrative of the typically zoonotic spread of the virus—81 human infections occurred after contact with imported infected rodents and subsequently infected pet prairie dogs but without evidence of subsequent human to human transmission.^22^, ^23^

Discontinuation of smallpox vaccination worldwide from the 1980s, which partially protects from infection with the antigenically related MPXV, may have contributed to recent reports of MPXV outbreaks in Central and West Africa,^10^, ^24^, ^25^, ^26^ including 188 suspected or confirmed cases in Nigeria in 2017^27^, ^28^ where human‐to‐human transmission in this and other outbreaks was proposed.^29^, ^30^, ^31^ More recently, often ongoing and larger outbreaks of MPXV have been documented in Central and West African countries particularly Cameroon, Central African Republic, and the Congo, with several infection‐related fatalities.^9^, ^27^, ^32^

Human travel‐related importations of MPXV to non‐African countries such as the UK have been occasionally reported.^33^, ^34^ However, the scale of the current outbreak of MPXV raises the possibility of potentially wider and sustained spread of MPXV outside of its African source area. Its suspected capability for human‐to‐human transmission in UK and European outbreaks in 2022 makes for the alarming possibility that MPXV infections may become indigenously established in human populations worldwide. An ability of MPXV to adapt to and efficiently transmit between humans in this outbreak situation is a critical determining factor for this possibility.

## 3 MPXV‐ASSOCIATED HUMAN DISEASE

MPXV infections are initially manifested by a non‐specific prodrome of fever, myalgia, lymphadenopathy, fatigue and headache 1–2 weeks after exposure, accompanied by the development of a characteristic centrifugal rash often becoming disseminated and affecting palms and soles of the feet.^35^, ^36^ The rash is initially maculopapular that proceeds to the development of vesicles and scabbing over a period of 12 days. Infectious virus is shed throughout the period of rash and in the prodromal period of systemic infection. Disease severity and the extent of rash formation is highly variable between individuals although more severe in those co‐infected with HIV‐1.^29^ Incubation and disease severity has been reported to vary by route of transmission; infection from percutaneous routes, such as bites and scratches from infected prairie dogs, showed greater severity of systemic symptoms, higher likelihood of hospitalisation and shorter incubations periods compared to those infected through close contact or respiratory routes.^37^ Greater systemic disease severity may be of relevance for those infected by viraemic blood component transfusions or transplant, where relatively large virus amounts may be introduced systemically.

There is little or no information on disease severity in highly immunocompromised individuals, although these may follow the severe and often potentially fatal outcomes of other transfusion‐transmitted infections of human cytomegalovirus, and hepatitis B and E viruses in these patients. This should be considered for transplant and transfusion safety given the high proportion of immunocompromised recipients of blood, platelets and organs. The existence of residual immune protection from vaccinia vaccination may, however, ameliorate disease severity in patients over 50.

Detailed clinical and virological characterisation of MPXV infection in seven young individuals without comorbidities demonstrated a highly variable course of infection and infection markers,^4^ in terms of the occurrence of rash and observational period relative to time from exposure. However, six from seven showed often prolonged periods of viraemia (up to 30 days) and excretion of MPXV in respiratory samples and urine.

## 4 DIAGNOSIS AND CASE DEFINITIONS

Clinical diagnosis of MPXV infections may be compounded by marked differences in severity and extents of spread of rash. The European Centre for Disease Control (ECDC) has proposed case definitions of “Confirmed” on the basis of a positive result in a PCR specific for MXPX or for orthopoxviruses and sequence confirmation.^3^ “Probable” cases are defined as those with rash, one from a range of associated disease features typical of MPXV and epidemiological links to diagnosed MPXV cases, travel to endemic countries, being an MSM, having multiple sexual partners or positivity in an orthopoxvirus‐specific PCR without demonstration that it is MPXV. Case definitions for possible, probable and confirmed MPX published by UKHSA are comparable,^2^ with probable cases defined as “a person with an unexplained rash on any part of their body plus one or more classical symptom or symptoms of monkeypox infection since March 15, 2022 and either: has an epidemiological link to a confirmed or probable case of monkeypox in the 21 days before symptom onset; or reported a travel history to west or central Africa in the 21 days before symptom onset; or is a gay, bisexual or other man who has sex with men.” The “probable” case definition clearly lacks sensitivity and many laboratory‐confirmed cases of MPX would fall outside this description particularly with increasing transmission within the community.^38^

While nucleic acid tests (NATs) for MPXV DNA provide a definitive diagnosis of MPXV infection, there are currently no commercially available MPXV molecular assays in Europe although the GenXpert PCR‐based assay has been widely used in Africa.^39^ Testing in Europe currently requires a laboratory to perform in‐house PCRs and access to positive and negative control materials for assay validation. Alternative methods such as electron‐microscopy, isolation and tissue immunocytochemistry for MPXV virus detection are less sensitive^40^ and impractical for large‐scale routine diagnostic purposes. Networks of collaborating laboratories for screening and reference testing for MPXV are established in many European countries and by UKHSA, but testing is currently on far too small a scale to enable routine diagnosis of infection and operates at a fraction of the scale required to monitor transfusion and transplant safety (discussed below). Limited testing capacity, and difficulties with clinical ascertainment of cases makes substantial underestimation of the scale of the current outbreaks in Africa and Europe more than likely.

## 5 EMERGENCE OF MPXV IN THE UK AND EUROPE

A travel‐related infection (from Lagos, Nigeria) was reported by UKHSA on the 7th May 2022, but six subsequent confirmed cases and one probable case were reported on the 13th–15th May in individuals without travel histories to Africa and no evident links to imported cases.^41^ Four of these were MSMs as were a further two diagnosed on the 18th May, and a further 11 on the 20th May. At time of writing (10th July, 2022) the latest available UK total stands at 1552 confirmed cases^1^ (Figure 1). Reporting from elsewhere in Europe between the in early July described 4913 cases, primarily in GBMSMs. Rapidly increasing numbers of cases have also been reported in in Canada and the US (1125 cases on the 8th July). MPXV infections remain strongly associated with GBMSMs with shared risk factors for HIV‐1 infections and other sexually transmitted infections (STIs). An enhanced surveillance questionnaire by UKHSA^1^ identified 96% of reported MPX cases in the UK to be in GBMSMs, 54% with a history of STI in the previous year and use of pre‐exposure prophylaxis for HIV‐1 in nearly 80% of those uninfected with HIV‐1. A comparison of the epidemiological and clinical features of pre‐ and post‐outbreak MPXV infections is summarised in Table 1.

**TABLE 1:** Reported cases of MPXV infections associated with the current outbreak

	Pre‐outbreak MPX	Outbreak MPX
Source	Suspected wide distribution in rodents in sub‐Saharan Africa	Unknown
Human Infections	Sporadic infections in Central / West Africa, travel‐related introductions into Europe, USA and elsewhere	Infections primarily among GBMSMs in UK, elsewhere in Europe and North America
Transmission	Inefficient between humans; infections primarily zoonotic	Sustained chains of human‐to‐human transmission by close or sexual contact
Disease presentations	Prodromal systemic symptoms, including lymphadenopathy, pyrexia, myalgia. Centrifugal maculopapular rash proceeding to vesicle formation	Prodromal systemic symptoms common, perianal or rectal lesions often resembling other STIs.
Morbidity and Mortality	High—10‐15% mortality in cases in Central Africa (clade 1 MPXV); around 1% mortality for clades 2, 3, (West Africa)	Low—10% hospitalisation rate primarily for symptom control. No MPX‐associated deaths recorded in Europe.
Demographics	All ages, male, female	Primarily males; age range 20–55
Risk factors for infection	Travel from endemic area (Central, West Africa), contact with infected rodents	Close or sexual contact with GBMSMs; shared risk factors for HIV‐1 infection
Virus genetics	MPXV strains genetically diverse—Clade 1 (central Africa), clades 2, 3 (West Africa)	Minimal genetic variability—point source for all cases to date; multiple potential APOBEC related mutations.

All MPXV strains genetically characterised in the current outbreak belong to clade 3, part of the “West African” clade found in Cameroon and Sierra Leone.^42^ Importantly, this clade shows a lower (<1%) case‐fatality ratio (CFR) than clade 1 viruses found in Central Africa with a CFR of 10% or greater.^43^, ^44^ Consistent with their genetic affiliation, infections of MPXV in the current outbreak have been mild, with no deaths from MPXV infection in the UK and 3 from 6027 cases reported worldwide from the current outbreak.^1^ Approximately 10% of UK cases have been hospitalised although some instances are for containment reasons.

Notwithstanding the difficulties in diagnosis and likely substantial under‐reporting of cases, the current global MPXV outbreak is unprecedented. Its spread may be facilitated by greater travel in the COVID‐19 post‐pandemic period and through a growing reduction in population immunity following the cessation of vaccinia vaccination for smallpox. Alternatively, there may be genetic adaptations among currently spreading strains of MPXV that facilitate its human‐to‐human spread. The latter hypothesis is supported by current (and still preliminary and incomplete) genetic analysis of currently sequenced strains of MPXV. While the estimated substitution rate of the related vaccinia virus is of the order of 1–2 mutations across a genome of over 150 000 base pairs per year, current outbreak strains are genetically highly homogeneous, consistent with a very recent point source origin for UK strains and subsequent spread into other European countries and North America.^42^, ^45^

Intriguingly, outbreak associated MPXV sequences also contain a large number of C‐>U or G‐>A mutations,^42^, ^45^ thought to be introduced by the action of nucleic acid editing proteins (APOBECs) on positive or negative DNA strands of the MPXV genome. This cellular defence mechanism reduces the replication of susceptible viruses through the introduction of mutations into the virus genome during its replication.^46^ Such mutational biases are not typically observed among MPXV strains infecting rodents, other mammals or indeed human infection directly derived from reservoir sources and it appears that they may originate early in human‐to‐human transmission chains before the reported emergence of MPXV. Its adaptation for efficient human‐to‐human transmission may ultimately require the evolution of more effective defences against this and potentially many other host‐specific defence pathways. Given the scale of the current outbreak and the rapid increase in reported cases in the UK and elsewhere (Figure 1), perhaps this has already been achieved.

## 6 EVALUATION OF THE RISK OF TRANSFUSION‐ AND TRANSPLANT‐ASSOCIATED TRANSMISSION

The risk of transmission of MPXV by substances of human origin (SOHO) for recipients in the EU/EEA area has been evaluated by the European Center for Disease Control,^3^, ^47^ While it recognises that monkeypox virus is likely to be transmissible through SOHOs, the overall risk is estimated to be low, for reasons of a historical absence of documented transmission of this virus from such sources, uncertainty about the duration of viremia and a lack of data on the duration and viral loads of MPXV in asymptomatic patients. In the UK, the UK Standing Advisory Committee on Transfusion Transmitted Infections (SACTTI) has produced a position statement on MPXV infection in donors and its potential transmissibility. The Joint United Kingdom (UK) Blood Transfusion and Tissue Transplantation Services Professional Advisory Committee (JPAC) have subsequently issued guidance on donor selection, specifically that donors with recently diagnosed MPXV infections cannot donate within 28 days nor can close contacts of individuals with MPXV infection within the last 21 days.^48^ Questions to ascertain MPXV exposure have been added to donor interview in the UK and elsewhere.

Our brief review of available information about the current MPXV outbreak and has highlighted several areas to be considered in an assessment of the possibility of transfusion and transplant‐associated transmission of the virus from donors with undiagnosed MPXV infections. Reduction of virus transmission risk relies on donor selection to avoid high‐risk sexual or other behaviours associated with HIV and other blood‐borne infections and through highly sensitive screening for defined transfusion‐transmissible pathogens (including HIV, hepatitis B, C and E viruses). In the absence of specific screening for MPXV, the most important factors contributing to likelihood of transmission are therefore the frequency of actively infected donors, the amount of infectious virus in blood or organs, retained virus infectivity during component manufacture and storage, the overlap of risk behaviours in donors associated with MPXV infections with those of other pathogens and the susceptibility of blood, platelet and transplant donations to severe MPXV‐induced disease.

Information on these variables is currently incomplete and often speculative. However, several factors contribute to the authors' concern about the risk that MPXV poses for transfusion and transplant safety:

- While the current number of report of MPXV infections is relatively low, they are rapidly increasing at the time of writing^1^, ^49^ and showing a maintained doubling time of around 2 weeks in the UK and elsewhere (Figure 1). Given the lag between infection, diagnosis and case reporting, the frequency of active infections in the wider community in the UK and elsewhere in Europe may be considerably higher than currently reported.
- Current investigations of infection outcomes of MPXV are only described for those presenting with symptomatic infection (and then only in the latter stages of the infection cycle). The extent of virus replication and potential transmissibility in prodromal stages of infection or those with asymptomatic or undiagnosed infections,^50^ collectively representing those most likely to donate, are unknown. For many other viruses such as HIV‐1, hepatitis B, and C viruses, however, viral loads are highest before symptom or disease onset while even those with asymptomatic infections may display prolonged viraemia throughout what is a relatively long period of virus replication (up to 30 days). Combined, these factors suggest that that blood component and organ donations from donors with entirely inapparent MPXV infections may nevertheless be viraemic.
- What dose of MPXV might be infectious by transfusion or transplant is unknown. For example, MPXV virions may be immune‐complexed and potentially neutralised by anti‐MPXV antibodies in blood or tissues, although this might typically reduce infectivity only in later stages of infection post‐seroconversion for antibodies. As described, donors are more likely to donate when in an early prodromal stage of infection where viraemia levels may be actually higher than recorded in those with symptomatic infections (see above).
- Under the recently introduced FAIR guidelines for donor selection, those who disclose performing anal sex within the previous 3 months with a new or multiple partners cannot donate, and this would therefore exclude many individuals currently identified in these MPXV outbreak investigations in the UK. A travel history to Central or West Africa would similarly defer donors for four or more months from donating. While these donor selection criteria will substantially reduce transmission risk, ongoing spread of MPXV outside the currently identified main risk groups for infection may hamper the future identification of risk factors for infection (as encapsulated by the likely relatively insensitive criteria used by UKHSA and ECDC to detect “possible” or “probable” infections). These include men who do not identify as MSM as they are less likely to be in contact with genitourinary medicine services.
- If MPXV adapts to humans and become more transmissible, it may then become endemic; current associations with defined risk behaviours will progressively weaken making identification of infected donors problematic and increasingly non‐specific. Based on current data, MPXV may already be substantially adapted for human‐to‐human transmission, in which case, the current case numbers will be largely underestimated.
- Although some MPXV strains in Central Africa show case fatality rates of greater than 10%,^44^, ^51^ infections in the UK and elsewhere associated with the current outbreak have been so far typically mild and spontaneously resolving, with no MPX‐associated deaths to date and a low hospitalisation rate.^1^ There is, however, a likelihood of much more severe disease outcomes in recipients of blood components and organ transplant. Firstly current data indicates greater disease severity in individuals infected through percutaneous routes.^37^ Secondly, blood component and transplant recipients are likely to be generally much more susceptible to severe disease from immunosuppression and severe intercurrent disease.

NHSBT and other UK blood services subscribe to the ABO Risk‐Based Decision‐Making Framework for Blood Safety and recognise that blood transfusion cannot be zero‐risk. In this context, broader strategies that inform the Department of Health and Social Security are provided by the Advisory Committee on the Safety of Blood, Tissues and Organs for blood services in the UK. However, we believe that the currently unquantifiable but likely real risk of MPXV transmission and the greater susceptibility of infections in blood and transplant recipients should prompt further urgent consideration. An important element in evaluating risk could be establishing a mechanism to rapidly determine viraemia frequencies in donors. This would provide much more direct data on transmission risk that would complement evaluations of projected infection incidences, risk behaviour and donor selection criteria that are essential parts of current risk assessments.

## 7 STRATEGIES FOR PREVENTION OF TRANSMISSION OF EMERGING PATHOGENS

We consider that a second testing channel within the blood services premises that can be activated at short notice for testing for additional agents (in this case MPXV) would be highly advantageous and be of major longer term strategic value for blood safety. Activation of such testing might be triggered through initial unlinked anonymised testing of several tens of thousands of donation samples to establish viraemia frequencies. The data might then justify taking no action with reassurance, implementing selective screening or alternatively urgent adoption of universal screening and exclusion of donations positive for the new agent. The rapid identification, recall and clinical assessment of donors identified in this way would be of medical value to those identified, and have substantial importance for national surveillance, not least in providing the means to identify donors most at risk for infection.

Identification of infected individuals would furthermore allow investigation of secondary transmission, wider contact tracing and ultimately prevention. Establishing a second channel testing capability within the blood services infrastructure would have to resourced nationally although it would be assisted considerably by recently advances in automated extraction methods, robotics for PCR setup and associated laboratory information management systems. Putting this into a wider context, such testing would be dwarfed in scale compared to the testing implemented for SARS‐CoV‐2 in UK government testing laboratories during the COVID‐19 pandemic. Even universal screening of blood and platelet donors in England would entail testing of five to six hundred pooled samples per week compared to up to 100 000 samples per day for SARS‐CoV‐2.

While the extent of MPXV emergence is currently limited and may ultimately subside, the expansion of the testing infrastructure so described would function as an important element in pandemic preparedness; SARS‐CoV‐2 was not in the end a blood‐borne pathogen but the next one may be and an inability to implement a short‐term response testing framework may have severe consequences for blood safety in the future.

## AUTHOR CONTRIBUTIONS

PS and HH conceived the reveiw and both contributed to the literature review, writing, review and journal submission.

## CONFLICT OF INTEREST

The authors declare no competing interests.
